# Efficacy and feasibility of proton beam radiotherapy using the simultaneous integrated boost technique for locally advanced pancreatic cancer

**DOI:** 10.1038/s41598-020-78875-1

**Published:** 2020-12-10

**Authors:** Tae Hyun Kim, Woo Jin Lee, Sang Myung Woo, Eun Sang Oh, Sang Hee Youn, Hye Young Jang, Sung-Sik Han, Sang-Jae Park, Yang-Gun Suh, Sung Ho Moon, Sang Soo Kim, Dae Yong Kim

**Affiliations:** 1 Center for Liver and Pancreatobiliary Cancer, National Cancer Center, Goyang, 10408 Republic of Korea; 2 Center for Proton Therapy, National Cancer Center, Goyang, 10408 Republic of Korea; 3 Department of Radiology, National Cancer Center, Goyang, 10408 Republic of Korea; 4 Biostatistics Collaboration Team, Research Core Center, National Cancer Center, Goyang, 10408 Republic of Korea

**Keywords:** Cancer therapy, Oncology, Cancer, Gastrointestinal cancer, Pancreatic cancer, Cancer, Radiotherapy

## Abstract

To evaluate the clinical efficacy and feasibility of proton beam radiotherapy (PBT) using the simultaneous integrated boost (SIB) technique in locally advanced pancreatic cancer (LAPC), 81 LAPC patients receiving PBT using SIB technique were analyzed. The prescribed doses to planning target volume (PTV)1 and PTV2 were 45 or 50 GyE and 30 GyE in 10 fractions, respectively. Of 81 patients, 18 patients received PBT without upfront and maintenance chemotherapy (group I), 44 received PBT followed by maintenance chemotherapy (group II), and 19 received PBT after upfront chemotherapy followed by maintenance chemotherapy (n = 16) (group III). The median follow-up time was 19.6 months (range 2.3–57.6 months), and the median overall survival (OS) times of all patients and of those in groups I, II, and III were 19.3 months (95% confidence interval [CI] 16.8–21.7 months), 15.3 months (95% CI 12.9–17.7 months), 18.3 months (95% CI 15.9–20.7 months), and 26.1 months (95% CI 17.8–34.3 months), respectively (*p* = 0.043). Acute and late grade ≥ 3 toxicities related to PBT were not observed. PBT with the SIB technique showed promising OS for LAPC patients with a safe toxicity profile, and intensive combinations of PBT and chemotherapy could improve OS in these patients.

## Introduction

Various treatment options, including chemotherapy alone, chemoradiotherapy (CRT), chemotherapy followed by CRT/radiotherapy (RT), and CRT/RT, have been applied in locally advanced pancreatic cancer (LAPC) patients^1^. Recent randomized trials have shown the superiority of intensive chemotherapeutic regimens over gemcitabine alone in metastatic pancreatic cancer with good performance status (PS)^2,3^, and several studies have also shown promising outcomes in LAPC patients^4,5^. However, many patients have difficulty receiving and continuing these intensive treatments due to the high incidence of severe toxicity^2,3^. Although distant metastasis is a major cause of disease progression in LAPC patients, local disease control is also clinically important because approximately 30–40% of pancreatic cancer patients die without evidence of distant metastasis^6–8^. In addition, in the LAP07 trial comparing CRT after induction chemotherapy with chemotherapy alone^9^, CRT showed significant benefits in prolonging the period without treatment and the time to local progression. Thus, although the role of RT has been controversial due to conflicting results from clinical trials^9,10^, RT could still be one of acceptable treatment options for LAPC patients.

When administering RT with X-rays in LAPC patients, the proximity of the pancreatic tumor to gastrointestinal (GI) organs and inherent physical properties of X-rays limit the ability to deliver higher doses to the tumor(s) while minimizing the doses to surrounding normal tissues including GI organs; moreover, the high incidence of GI toxicity is one of the limiting factors^9–18^. Because of its unique physical properties, proton beams can deliver high doses to the target(s) without an exit dose outside the target(s), and proton beam radiotherapy (PBT) has shown safe and promising outcomes in various tumors^19–30^. In addition, PBT using the simultaneous integrated boost (SIB) technique can simultaneously deliver higher doses to the tumor(s) and lower doses to surrounding normal tissues to improve the therapeutic ratio, reduce the overall treatment time and minimize chemotherapy breaks. Thus, PBT using the SIB technique with various schedules and regimens of chemotherapy depending on the patient’s PS and age has been applied for LAPC patients in our institution. The aim of this study was to evaluate the clinical efficacy and feasibility of PBT using SIB technique and the combination effect of PBT and chemotherapy in LAPC patients.

## Results

A total of 106 patients treated with PBT between June 2013 and June 2019 were registered. Of these, 25 patients did not meet the inclusion criteria for the following reasons: 13 patients had T3 disease, 7 had locoregional recurrent disease after surgical resection, 4 had disease with other histologic types (i.e., neuroendocrine carcinoma and acinar cell carcinoma), and 1 had distant metastasis. The remaining 81 patients were analyzed in this study (Table 1). The time and regimens of chemotherapy before, during, and after PBT were chosen by the physicians considering the patient’s PS and age. Of the 81 patients, 18 (22.2%) patients received PBT without upfront and maintenance chemotherapy (group I), 44 (54.3%) patients received PBT followed by maintenance chemotherapy (group II), and 19 (23.5%) patients received PBT after upfront chemotherapy prior to PBT (group III). In group I (n = 18), 13 (72.2%) patients received concurrent chemotherapy; in group II (n = 44), 42 (95.5%) patients received concurrent chemotherapy. In group III (n = 18), the median cycles of chemotherapy before PBT and the interval from the start of chemotherapy to PBT were 7 cycles (range 2–18) and 6.4 months (range 1.8–18.0), respectively; moreover, 17 (89.5%) and 16 (84.2%) patients received concurrent and maintenance chemotherapy, respectively. The distributions of patients ≥ 70 years old and 60–69.9 years old were significantly higher in group I, followed by group II and group III, and the distribution of the patients with PS 1 was also significantly higher in group I than in groups II and III (*p* < 0.05 each) (Table 1). The radiation doses to the PTV1 were 45 GyE in 61 (75.3%) patients and 50 GyE in 20 (24.7%) patients, and there were no significant differences among groups I, II and III.

**Table 1:** Patient characteristics.

Characteristic	All, n (%)	Group I, n (%)	Group II, n (%)	Group III, n (%)	*p*-value
**Sex**					
Male	45 (55.6)	6 (33.3)	27 (61.4)	12 (63.2)	0.098^‡^
Female	36 (44.4)	12 (66.7)	17 (38.6)	7 (36.8)	
**Age (years)**					
Median (range)	66 (44–92)	76 (58–92)	66 (48–81)	58 (44–81)	< 0.001^∥^
< 60	23 (28.4)	1 (5.6)	11 (25.0)	11 (57.9)	< 0.001^§^
60–69.9	27 (33.3)	3 (16.7)	18 (40.9)	6 (31.6)	
≥ 70	31 (38.3)	14 (77.8)	15 (34.1)	2 (10.5)	
**ECOG PS**					
0	72 (88.9)	13 (72.2)	41 (93.2)	18 (94.7)	0.039^§^
1	9 (11.1)	5 (27.8)	3 (6.8)	1 (5.3)	
**Histology**					
Adenocarcinoma	81 (100)	18 (100)	44 (100)	19 (100)	1.000^§^
**Tumor location**					
Head/neck	49 (60.5)	12 (66.7)	28 (63.6)	9 (47.4)	0.399^‡^
Body/tail	32 (39.5)	6 (33.3)	16 (36.4)	10 (52.6)	
Tumor size* (cm)					
Median (range)	3.7 (2.2–7.3)	3.5 (2.2–5.5)	3.7 (2.2–7.3)	3.8 (2.7–6.2)	0.384^∥^
< 4	47 (58.0)	12 (66.7)	25 (56.8)	10 (52.6)	0.669^‡^
≥ 4	34 (42.0)	6 (33.3)	19 (43.2)	9 (47.4)	
T classification					
T4	81 (100)	18 (100)	44 (100)	19 (100)	1.000^§^
**N classification**					
N0	60 (74.1)	13 (72.2)	30 (68.2)	17 (89.5)	0.228^§^
N+	21 (25.9)	5 (27.8)	14 (31.8)	2 (10.5)	
**Pretreatment CA 19-9 level (U/mL)**					
Median (range)	43.6 (2–4740)	103.0 (2–4740)	45.1 (5–4630)	27.7 (5–984)	0.308^∥^
≤ 37	38 (46.9)	7 (38.9)	21 (47.7)	10 (52.6)	0.695^‡^
> 37	43 (53.1)	11 (61.1)	23 (52.3)	9 (47.4)	
**Pre-PBT CA 19-9 level (U/mL)**					
Median (range)	41.3 (2–4740)	103.0 (2–4740)	45.1 (5–4630)	20.9 (5–758)	0.275^∥^
≤ 37	39 (48.1)	7 (38.9)	21 (47.7)	11 (57.9)	0.511^‡^
> 37	42 (51.9)	11 (61.1)	23 (52.3)	8 (42.1)	
**Post-PBT CA 19-9 level (U/mL)**					
Median (range)	36.5 (2–4980)	41.6 (2–1885)	56.1 (5–4980)	19.6 (5–1310)	0.795^∥^
≤ 37	41 (50.6)	9 (50.0)	20 (45.5)	12 (63.2)	0.434^‡^
> 37	40 (49.4)	9 (50.0)	24 (54.5)	7 (36.8)	
**Chemotherapy prior to PBT**					
No	62 (76.5)	18 (100)	44 (100)	0 (0)	< 0.001^§^
Yes	19 (23.5)	0 (0)	0 (0)	19 (100)	
FOLFIRINOX	9 (11.1)	–	–	9 (47.4)	
GA	4 (4.9)	–	–	4 (21.1)	
GT	3 (3.7)	–	–	3 (15.8)	
GX	2 (2.5)	–	–	2 (10.5)	
GP	1 (1.2)	–	–	1 (5.3)	
**Concurrent chemotherapy**					
No	9 (11.1)	5 (27.8)	2 (4.5)	2 (10.5)	0.025^§^
Yes	72 (89.5)	13 (72.2)	42 (95.5)	17 (89.5)	
Capecitabine	70 (86.4)	12 (66.7)	41 (93.2)	17 (89.5)	
5-Fluorouracil	2 (2.5)	1 (5.6)	1 (2.3)	0 (0)	
**Maintenance chemotherapy**					
No	21 (25.8)	18 (100)	0 (0)	3 (15.8)	< 0.001^§^
Yes	60 (74.1)	0 (0)	44 (100)	16 (84.2)	
GA	15 (18.5)	–	13 (29.5)	2 (26.3)	
FOLFIRINOX	14 (17.3)	–	9 (20.5)	5 (26.3)	
Gemcitabine	11 (13.6)	–	7 (15.9)	4 (21.1)	
GT	10 (12.3)	–	8 (18.2)	2 (10.5)	
S-1	9 (11.1)	–	7 (15.9)	2 (10.5)	
OX	1 (1.2)	–	0 (0)	1 (5.3)	
**Post-PBT surgery**^†^					
No	77 (95.1)	17 (94.4)	41 (93.2)	19 (100)	0.654^§^
Yes	4 (4.9)	1 (5.6)	3 (6.8)	0 (0)	
**Radiation dose (GyE)**					
45	61 (75.3)	16 (88.9)	34 (77.3)	11 (57.9)	0.089
50	20 (24.7)	2 (11.1)	10 (22.7)	8 (42.1)	
**Overall tumor response**					
Partial response	5 (6.2)	1 (5.6)	3 (6.8)	1 (5.3)	0.933^§^
Stable disease	65 (80.2)	15 (83.3)	36 (81.8)	14 (73.7)	
Progressive disease	11 (13.6)	2 (11.1)	5 (11.4)	4 (21.1)	
**Primary tumor response**					
Partial response	24 (29.6)	6 (33.3)	15 (34.1)	3 (15.8)	0.355^§^
Stable disease	57 (70.4)	12 (66.7)	29 (65.9)	16 (84.2)	
Progressive disease	0 (0)	0 (0)	0 (0)	0 (0)	
**Median follow-up time (months)**					
Median (range)	19.6 (2.3–57.6)	15.8 (2.3–52.0)	17.2 (3.9–54.1)	21.8 (7.6–57.6)	0.083^∥^

*ECOG PS* Eastern Cooperative Oncology Group performance status, *N+* lymph node positive, *CA 19-9* carbohydrate antigen 19-9, *SIB-PBT* simultaneous integrated boost-proton beam therapy, *FOLFIRINOX* 5-fluorouracil, irinotecan, and oxaliplatin, *GA* gemcitabine plus nab-paclitaxel, *GT* gemcitabine plus erlotinib, *GX* gemcitabine plus capecitabine, *GP* gemcitabine plus cisplatin, *S-1* tegafur-gimeracil-oteracil potassium, *OX* oxaliplatin plus capecitabine, *GyE* Gray equivalent.

*Maximum diameter of the primary tumor.

^†^Pylorus-preserving pancreaticoduodenectomy.

^‡^Pearson’s chi-square test.

^§^Fisher’s exact test.

^∥^One-way analysis of variance.

**Figure 1:**
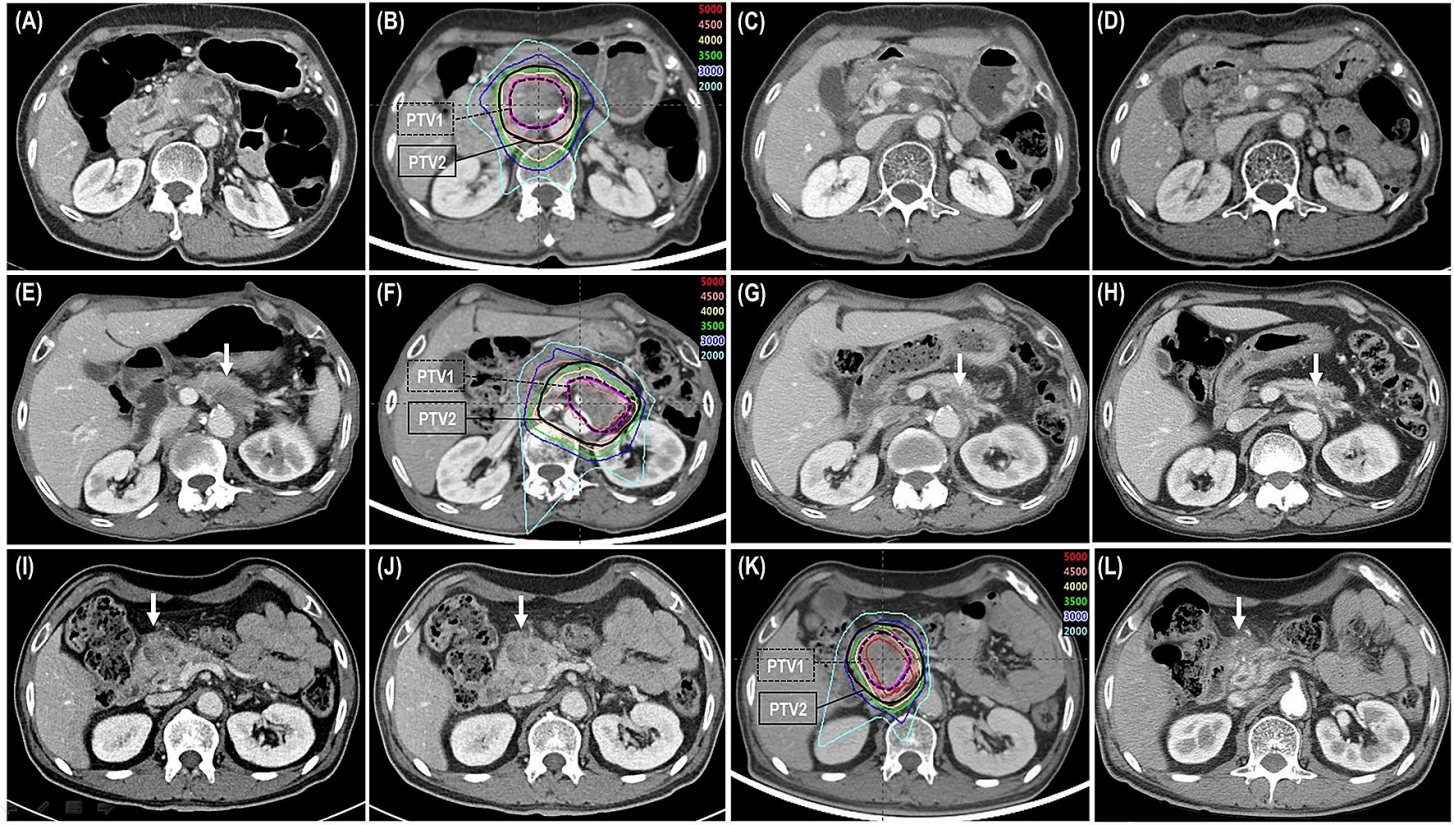
Partial response (PR) of a primary tumor after proton beam radiotherapy (PBT) in groups I (**A**–**D**), II (**E**–**H**), and III (**I**–**L**). In the group I: (**A**) initial CT scans showing the primary tumor (arrow); (**B**) the patient received PBT with concurrent capecitabine; (**C**,**D**) CT scans 4 and 7 months after PBT showing the PR of the primary tumor (arrow). In the group II: (**E**) initial CT scans showing the primary tumor (arrow); (**F**) the patient received PBT with concurrent capecitabine followed by maintenance chemotherapy with tegafur-gimeracil-oteracil potassium; (**G**,**H**) CT scans 1 and 4 months after PT showing the PR of the primary tumor (arrow). In the group III: (**I**) initial CT scans showing the primary tumor (arrow); (**J**) pre-PBT CT scans showing the primary tumor (arrow) after chemotherapy with 6 cycles of gemcitabine and nab-paclitaxel; (**K**) the patient received PBT with concurrent capecitabine followed by maintenance chemotherapy with oxaliplatin plus capecitabine; (**L**) CT scans 4 months after PBT demonstrating the PR of the primary tumor (arrow). *PTV* planning target volume.

After the completion of PBT, the overall and primary tumor responses were summarized in Table 1 (Fig. 1) and there were no significantly differences among groups I, II and III (*p* < 0.05 each). The primary tumor responses were also not significantly different between patients receiving 45 GyE and those receiving 50 GyE (partial response, 19 [31.1%] and 5 [25%]; and stable disease, 42 [68.9%] and 15 [75%], respectively, *p* = 0.413). Of 81 patients, 4 (4.9%) patients who had resectable disease after PBT and surgical resection with negative margins was performed in 1 (5.6%), 3 (6.8%), and 0 (0%) patients in groups I, II and III, respectively (*p* = 0.654) (Table 1).

**Figure 2:**
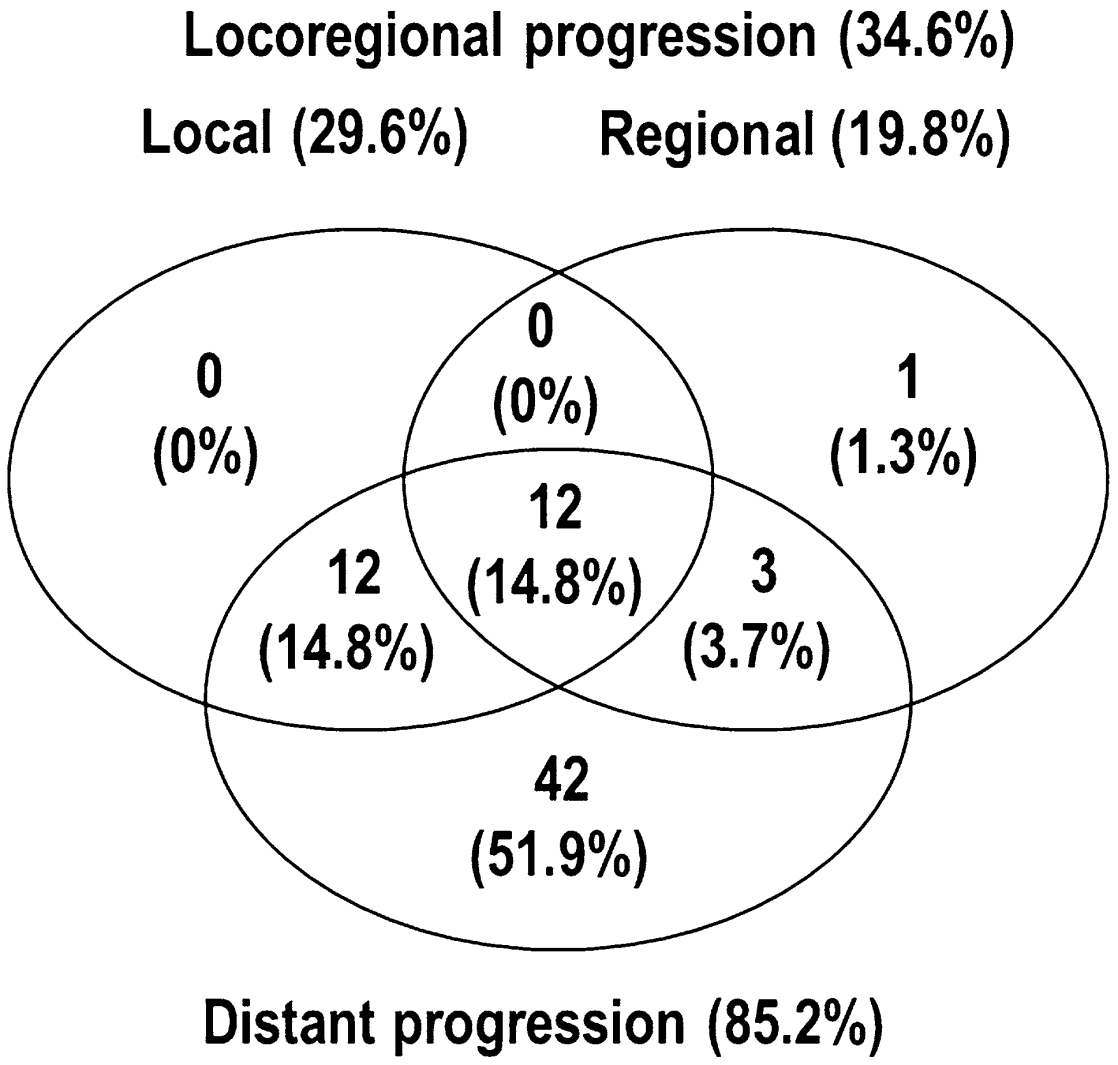
Patterns of disease progression.

At the time of analysis, 63 patients had died from the disease, and 18 remained alive. The median follow-up time and patterns of disease progression were summarized in Table 1 and Fig. 2. In all patients, median LRC times and 1-year LRC rates from the start of the first treatment and PBT were 19.2 months (95% CI 14.8–23.6 months) and 16.0 months (95% CI 11.8–20.3 months), respectively, and 79.4% (95% CI 70–88.8%) and 69.2% (95% CI 58.4–80%), respectively. The median PFS times from the start of the first treatment and PBT were 10.1 months (95% CI 7.5–12.6 months) and 9.0 months (95% CI 7.3–10.8 months), respectively, and the median OS times from the start of the first treatment and PBT were 19.3 months (95% CI 16.8–21.7 months) and 18 months (95% CI 15.5–20.5 months), respectively (Supplementary Fig. 1).

**Figure 3:**
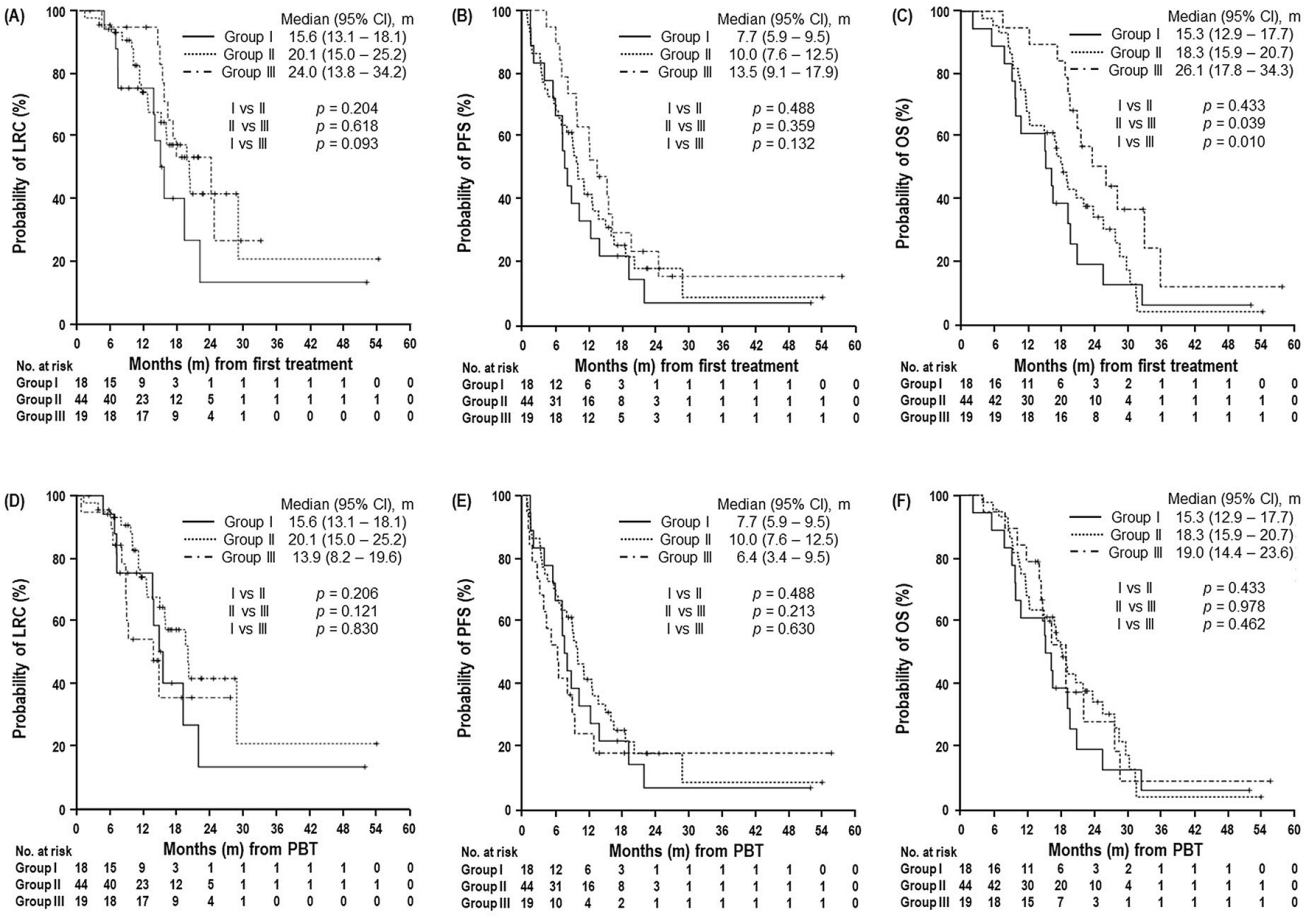
Locoregional control (LRC) (**A**), progression-free survival (PFS) (**B**), and overall survival (OS) (**C**) curves from initial treatment and LRC (**D**), PFS (**E**), and OS (**F**) from PBT.

Univariate analysis demonstrated that patient age, N classification, post-PBT surgery, and treatment group were significantly associated with OS (*p* < 0.05 each) (Table 2). Except for treatment group due to intercorrelation between treatment group and patient age (r = 0.5, *p* < 0.01), multivariate analysis showed that patient age, N classification and post-PBT surgery were significant factors independently associated with OS (*p* < 0.05 each) (Table 2). The OS from the start of the first treatment was significantly longer in group III, followed by group II, and group I (Table 2, Fig. 3C), and the PFS and LRC from the first treatment also tended to be longer in group III with the same order, but these differences were not significant (*p* > 0.05 each) (Fig. 3A,B). In addition, the OS and LRC from PBT tended to be longer in groups III and II than in group I, and the PFS from PBT tended to be longer in group II than in groups I and III without statistical significance (*p* > 0.05 each) (Fig. 3D–F).

**Table 2:** Univariate analysis of clinical characteristics associated with overall survival (OS).

Characteristic	Univariate		Multivariate	
	OS, median (95% CI), months	*p*-value*	Hazard Ratio (95% CI)	*p*-value^†^
**Sex**				
Male	19.1 (16.6–21.6)	0.786	–	
Female	19.7 (16.6–22.8)		–	–
**Age (years)**				
< 60	26.1 (23.4–28.7)	0.026	1.000	–
60–69.9	19.2 (16.4–22.0)		1.815 (0.915–3.601)	0.088
≥ 70	16.3 (10.4–22.2)		2.808 (1.583–5.315)	0.002
**ECOG PS**				
0	19.7 (10.6–28.6)	0.830	–	
1	19.3 (16.7–21.8)		–	–
**Tumor location**				
Head/neck	19.3 (16.6–22.0)	0.330	–	
Body/tail	18.8 (15.2–22.3)		–	–
Tumor size* (cm)				
< 4	19.3 (16.3–22.2)	0.443	–	
≥ 4	19.2 (15.8–22.7)		–	
**N classification**				
N0	20.7 (17.5–23.9)	0.018	1.000	–
N+	15.2 (8.5–21.8)		1.874 (1.082–3.245)	0.025
**Pretreatment CA 19-9 level (U/mL)**				
≤ 37	19.6 (14.3–24.9)	0.062	–	
> 37	19.1 (17.2–20.9)		–	–
**Pre-PBT CA 19-9 level (U/mL)**				
≤ 37	20.7 (16.6–24.8)	0.067	–	
> 37	18.8 (16.4–21.1)		–	–
**Post-PBT CA 19-9 level (U/mL)**				
≤ 37	19.6 (17.9–21.4)	0.129	-	
> 37	18.3 (14.0–22.6)		-	-
**Primary tumor response**				
Responder	23.8 (16.0–31.5)	0.226	–	
Nonresponder	18.8 (16.5–21.0)		–	
**Post-PBT surgery**				
No	19.1 (16.8–21.3)	0.037	1.000	–
Yes	31.4 (–)		0.136 (0.018–1.007)	0.051
**Radiation dose (GyE)**				
45	19.3 (16.3–22.2)	0.171	–	
50	23.6 (16.4–30.8)		–	–
**Concurrent chemotherapy**				
No	18.8 (3.3–34.2)	0.913	–	
Yes	19.3 (16.4–22.1)		–	–
**Treatment groups**				
I	15.3 (12.9–17.7)	0.043	–	
II	18.3 (15.9–20.7)		–	–
III	26.1 (17.8–34.3)		–	–

*Responder *complete or partial response, *Nonresponder *stable disease or progressive disease, *CI* confidence interval, and others are the same as in Table 1.

*Log rank test.

^†^Cox proportional hazards model.

PBT was completed without treatment interruption in all patients, and the distributions of acute toxicities are summarized in Table 3. The most common acute toxicities were grade 1 anemia (19.8%), grade 1 leukopenia (17.3%), grade 1 vomiting (12.3%) and grade 1 abdominal pain (8.6%); grade ≥ 3 acute toxicities were not observed. At the time of analysis, grade ≥ 3 late toxicities related to PBT, such as gastrointestinal ulcers or bleeding, were not detected.

**Table 3:** Acute toxicities during proton beam therapy.

Type of toxic effect	Grade 0, n (%)	Grade 1, n (%)	Grade 2, n (%)	Grade 3, n (%)	Grade 4, n (%)	Grade 5, n (%)
**Hematologic toxicity**						
Leukopenia	61 (75.3)	14 (17.3)	6 (7.4)	0 (0)	0 (0)	0 (0)
Anemia	64 (79.0)	16 (19.8)	1 (1.2)	0 (0)	0 (0)	0 (0)
Thrombocytopenia	76 (93.8)	5 (6.2)	0 (0)	0 (0)	0 (0)	0 (0)
**Nonhematologic toxicity**						
Hand-foot syndrome	81 (100)	0 (0)	0 (0)	0 (0)	0 (0)	0 (0)
Anorexia	74 (91.4)	4 (4.9)	3 (3.7)	0 (0)	0 (0)	0 (0)
Vomiting	66 (81,5)	10 (12.3)	5 (6.2)	0 (0)	0 (0)	0 (0)
Diarrhea	78 (96.3)	3 (3.7)	0 (0)	0 (0)	0 (0)	0 (0)
Abdominal pain	74 (91.4)	7 (8.6)	0 (0)	0 (0)	0 (0)	0 (0)
Stomatitis	79 (97.5)	1 (1.2)	1 (1.2)	0 (0)	0 (0)	0 (0)

Abbreviations: see Table 1.

Some patients experienced more than one toxicity.

National Cancer Institute Common Terminology Criteria for Adverse Events, version 4.0.

## Discussion

Chemotherapy and/or RT have been mainstays of treatment in LAPC patients. A recent meta-analysis^4^ of 11 studies including 315 LAPC patients initially treated with chemotherapy with the fluorouracil, irinotecan and oxaliplatin combination (FOLFIRINOX) and subsequently treated with CRT/RT (63.5%) and/or surgical resection (25.8%) showed a promising median OS of 24.2 months (range 10–32.7 months). However, a high rate of treatment-related adverse events, i.e., grade ≥ 3 toxicity of 60.4%, is an obstacle to the clinical application of these intensive treatments for LAPC patients. Traditionally, conventionally fractionated RT using a large RT volume with elective nodal irradiation has been used, but its toxicity was not trivial^9–11,17,18,31^. Due to recent technical advances in RT techniques, such as intensity-modulated RT (IMRT) and stereotactic body RT (SBRT), as well as the use of limited RT volumes without elective nodal irradiation, RT combined with chemotherapy has shown promising outcomes in terms of the median OS of 6–20 months and a favorable safety profile, i.e., grade ≥ 3 toxicity of 0–26%^12–15,32,33^. The dosimetric superiority of PBT compared to RT with X-rays has been reported in several studies^34,35^. Moreover, Nichols et al.^22^ analyzed 22 pancreatic and ampullary cancer patients treated with PBT (with 50–59.4 GyE in 28–33 fractions) and concurrent capecitabine and reported a safe toxicity profile, i.e., grade ≥ 3 GI toxicity of 0%. Clinical data on PBT for LAPC patients are still limited to date^21,25,27^. However, Terashima et al.^21^ analyzed 50 LAPC patients treated with PBT (with 50–70.2 GyE in 25–26 fractions) and concurrent gemcitabine and showed promising outcomes in terms of the 1-year OS and LRC rates (76.8% and 81.7%, respectively) and the grade ≥ 3 late toxicity rate (10%). Hiroshima et al.^28^ analyzed 42 LAPC patients who were treated with PBT (54–67.5 GyE in 25–33 fractions) and concurrent chemotherapy (100%), upfront chemotherapy before PBT (76.2%) and maintenance chemotherapy after PBT (81%) and reported a promising median OS of 27.5 months from initial treatment and grade ≥ 3 late GI toxicity of 0%. In the present study, we analyzed 81 LAPC patients treated with PBT with the SIB technique, prescribed 45–50 GyE or 30 GyE in 10 fractions to the target volumes according to the proximity of the GI organs, to improve the therapeutic ratio, reduce the overall treatment time and minimize chemotherapy breaks, and we observed a median OS of 19.3 months and no grade ≥ 3 acute and late toxicities.

Pancreatic cancer most frequently occurs in patients in their 60 s and 70 s^36,37^. With increased age, LAPC patients may be less likely to tolerate a prolonged course of intensive chemotherapy and/or RT, and less intensive anticancer treatments more frequently have been applied rather than intensive treatments in the real world^1^. Consistent with the clinical practice in the real world, in the present study, the proportion of patients ≥ 70 years old and/or with PS 1 was significantly high in group I (PBT ± concurrent chemotherapy), followed by group II (PBT ± concurrent chemotherapy + maintenance chemotherapy) and group III (upfront chemotherapy followed by PBT ± maintenance chemotherapy) (*p* < 0.05 each) (Table 1), and age was one of the significant prognostic factors associated with OS (Table 2). In addition, the median OS time from the start of the first treatment was significantly longer in group III, followed by group II and group I (26.1 months, 18.3 months, and 15.3 months, respectively, *p* = 0.043) (Table 2, Fig. 3C). The median PFS and LRC times from the first treatment also tended to be longer in group III, followed by group II and group I (13.5 months, 10 months, and 7.7 months; 24 months, 20.1 months, and 15.6 months, respectively, *p* > 0.05 each) (Fig. 3A,B). These findings suggested that age as well as the intensity of treatment could influence the clinical outcomes in terms of OS, PFS and LRC in LAPC patients. Recently, SBRT has been tried for LAPC patients to improve the therapeutic ratio, reduce the overall treatment time and minimize chemotherapy breaks^14,18,32,33^. Petrelli et al.^32^, in pooled analysis of 19 SBRT trials for borderline resectable and/or unresectable LAPC patients, reported a median OS time of 17 months, 1-year LRC rate of 72.3%, and grade ≥ 3 toxicities of < 10% and Mazzola et al.^33^, in analysis of 33 LAPC patients treated with risk-adapted SBRT and upfront chemotherapy before SBRT (72.7%), also showed favorable outcomes in terms of 1-year LRC and OS rates (81% and 75%, respectively) and grade ≥ 3 toxicity rate (0%). In the present study, the 1-year LRC rates and median OS times from the start of the first treatment and PBT were 79.4% and 69.2%, respectively, and 19.3 months and 18 months, respectively, and rate of grade ≥ 3 toxicity was 0%. Although direct comparisons of the present study with previous studies are not possible, the median OS time and rates of adverse events in LAPC patients treated with PBT with the SIB technique were on the upper and low ends, respectively, of the wide ranged results reported in previous studies^14,18,32,33^.

This study has several limitations. First, our data were from a single institutional nonrandomized retrospective study and various schedules and regimens of chemotherapy; thus, selection bias was not thoroughly evaluated. However, data of PBT for LAPC patients have been limited, at the best of our knowledge, present study included largest number of LAPC patients (n = 81) among the studies using PBT for LAPC patients until now. In addition, the sequences and regimens of chemotherapy were determined by the physicians, considering each patient’s tolerability, such as age and PS, and this reflects clinical practice in the real world. Thus, the present study has clinical significance, as it showed the possible combinations of PBT and/or chemotherapy and their effectiveness in the real world. Second, retrospective studies are likely to underreport adverse events due to the incompleteness of clinical notes, recall bias, etc., and several recent retrospective studies using modern RT techniques, such as IMRT and SBRT, have also shown favorable toxicity profiles^12–15,32,33^. Similar to the present study, PBT has also shown favorable toxicity profiles due to the dosimetric superiority of proton beams compared to X-rays^21,25,27^. In addition, PBT using the SIB technique with 10 fractions, as applied in the present study, can potentially improve the therapeutic ratio by escalating the radiation doses to the tumor(s) while minimizing the radiation doses to surrounding normal tissues and minimizing the chemotherapy break by reducing the overall treatment time. However, because there was no randomized study comparing PBT with RT with X-rays, including IMRT and SBRT, further large-scale comprehensive studies are needed.

In conclusion, the present study demonstrated that PBT with various sequences and regimens of chemotherapy for LAPC patients resulted in promising survival with a safe toxicity profile, and the patients treated with intensive combinations of PBT and chemotherapy showed improved OS. The median OS time was significantly longer in group III (upfront chemotherapy followed by PBT ± maintenance chemotherapy), followed by group II (PBT ± concurrent chemotherapy followed by maintenance chemotherapy) and group I (PBT ± concurrent chemotherapy). The present data suggest that PBT may be one of promising therapeutic options in LAPC patients and intensive combinations of PBT and chemotherapy, including upfront and maintenance chemotherapy, could consider to improving OS in these patients.

## Methods

### Patients

Patients who received PBT for pancreatic cancer between June 2013 and June 2019 were registered, and the database was reviewed. The inclusion criteria of the present study were as follows: (i) histologically proven adenocarcinoma of the pancreas; (ii) unresectable disease (stage cT4); (iii) no evidence of distant metastasis; and (iv) no previous or current uncontrolled malignancies outside of the pancreas. The American Joint Committee on Cancer (AJCC) staging classification (8th edition) was used for tumor staging. Unresectable disease (stage cT4) was defined as local tumor extension to the celiac axis, common hepatic artery, superior mesenteric artery, or superior mesenteric-portal venous confluence on multiphase computed tomography (CT) scans; lymph node positivity was defined as the presence of lymph node(s) of at least 1 cm in the short diameter, with an indistinct or speculated margin or with a mottled heterogenic pattern on CT and/or positron emission tomography (PET) scans^38^. This study was approved by the institutional review board (IRB) of the National Cancer Center (NCC) (NCC20200164) and all methods were performed in accordance with the relevant guidelines and regulations. The requirement for informed consent was waived by the IRB of NCC due to the retrospective nature of the study.

### Treatment

A contrast-enhanced four-dimensional CT scan was obtained for each patient. The internal target volume (ITV) and contours of organs at risk (OARs) were defined as the sum of the gross tumor volume(s) and each OAR during gated (exhalation) phases (30% of the total respiratory cycle), respectively, and the clinical target volume was regarded as the ITV with no additional margins for elective nodal basins^12,13,18,33^. The planning target volumes 1 and 2 (PTV1 and PTV2) were defined as the ITV plus 3–5 mm margins, excluding the 5 mm expanded volume of GI organs with the intention of avoiding GI toxicity, and the ITV plus 7–12 mm margins in all directions, respectively. The PBT plan with the SIB technique, described in detail previously^27^, was performed using two non- or coplanar beams of 230 meV passively double-scattered proton beams (Proteus 235; Ion Beam Applications, S.A., Louvain-la-Neuve, Belgium) encompassing the PTV2 and one beam encompassing the PTV1, and it was designed with the intention of delivering 100% of the prescribed doses to at least 90% of the PTVs. The radiation doses of PBT were expressed in Gray equivalents (GyE = proton physical dose [Gray] × relative biologic effectiveness [1.1]). The prescribed doses to the PTV1 and PTV2 were 45 or 50 GyE and 30 GyE in 10 fractions, respectively, 5 times a week (Fig. 1). The dose-volume constraints for the OARs were described previously^15,16,27^. In brief, the maximum dose to the spinal cord was less than 27 GyE; the absolute volumes of the stomach receiving ≥ 37 GyE and bowel receiving ≥ 35 GyE were less than 2 cm^3^; and the relative volumes of the liver receiving ≥ 27 GyE and kidney receiving ≥ 18 GyE were less than 60% and 35%, respectively. At each treatment, all patients were asked to fast for at least 4 h prior to PBT, and radiation was delivered during gated phases with a respiration-gated technique.

### Evaluation and statistical considerations

Clinical, laboratory and imaging assessments, such as physical examinations, complete blood counts, liver function tests, serum carbohydrate antigen (CA) 19-9, chest X-rays and abdominopelvic CT scans, were performed within 2 weeks before PBT, at the first month after PBT, every 3 months for the following 3 years and every 6 months thereafter. Tumor response was assessed according to the Response Evaluation Criteria in Solid Tumors criteria (version 1.1)^39^, and the response of the primary tumor was defined as the maximal tumor response observed during the follow-up period. Adverse events related to PBT were assessed using the Common Terminology Criteria for Adverse Events (version 4.03) (https://ctep.cancer.gov/protocolDevelopment/electronic_applications/docs/CTCAE_4.03.xlsx).

Disease progression was confirmed by pathologic and/or radiological findings showing an increase in size over time. Local, regional and distant progression were defined as progression of the primary tumor or recurrence at the primary tumor bed, progression in regional lymph nodes and soft tissues located near the primary tumor, and the development of distant metastasis, respectively. Locoregional control (LRC), progression-free survival (PFS) and overall survival (OS) were defined as the intervals from the date of the start of chemotherapy or PBT (whichever came first) to the date of locoregional progression, the date of any disease progression or death, and the date of death or the last follow-up, respectively^27^. The probabilities of survival were estimated using the Kaplan–Meier method and compared with the log rank test in univariate analysis. The hazard ratios (HRs) were estimated using the Cox proportional hazards model in multivariate analysis with a stepwise forward selection procedure. A *p* value of < 0.05 was considered statistically significant, and all statistical tests were performed with STATA software (version 14.0; StataCorp., College Station, TX, USA).

## Supplementary Information

Supplementary Information.
