# Supplementary material for: Efficacy and feasibility of proton beam radiotherapy using the simultaneous integrated boost technique for locally advanced pancreatic cancer
Source: Sci Rep. 2020 Dec 10;10:21712. doi: 10.1038/s41598-020-78875-1 (PMC7729854; doi:10.1038/s41598-020-78875-1)
Supplement: Supplementary file 1 — Supplementary Information. [file 41598_2020_78875_MOESM1_ESM.pdf]

Supplementary Information

Efficacy and Feasibility of Proton Beam Radiotherapy using the Simultaneous Integrated Boost Technique for Locally Advanced Pancreatic Cancer

Tae Hyun Kim[1,2*†], Woo Jin Lee[1†], Sang Myung Woo[1†], Eun Sang Oh[2], Sang Hee Youn[2], Hye Young Jang[3], Sung-Sik Han[1], Sang-Jae Park[1], Yang-Gun Suh[2], Sung Ho Moon[2], Sang Soo Kim[4], Dae Yong Kim[2]

[1]Center for Liver and Pancreatobiliary Cancer, National Cancer Center, Goyang, 10408, Republic of Korea

[2]Center for Proton Therapy, National Cancer Center, Goyang, 10408, Republic of Korea

[3]Department of Radiology, National Cancer Center, Goyang, 10408,Republic of Korea

[4]Biostatistics Collaboration Team, Research Core Center, National Cancer Center, Goyang, 10408, Republic of Korea

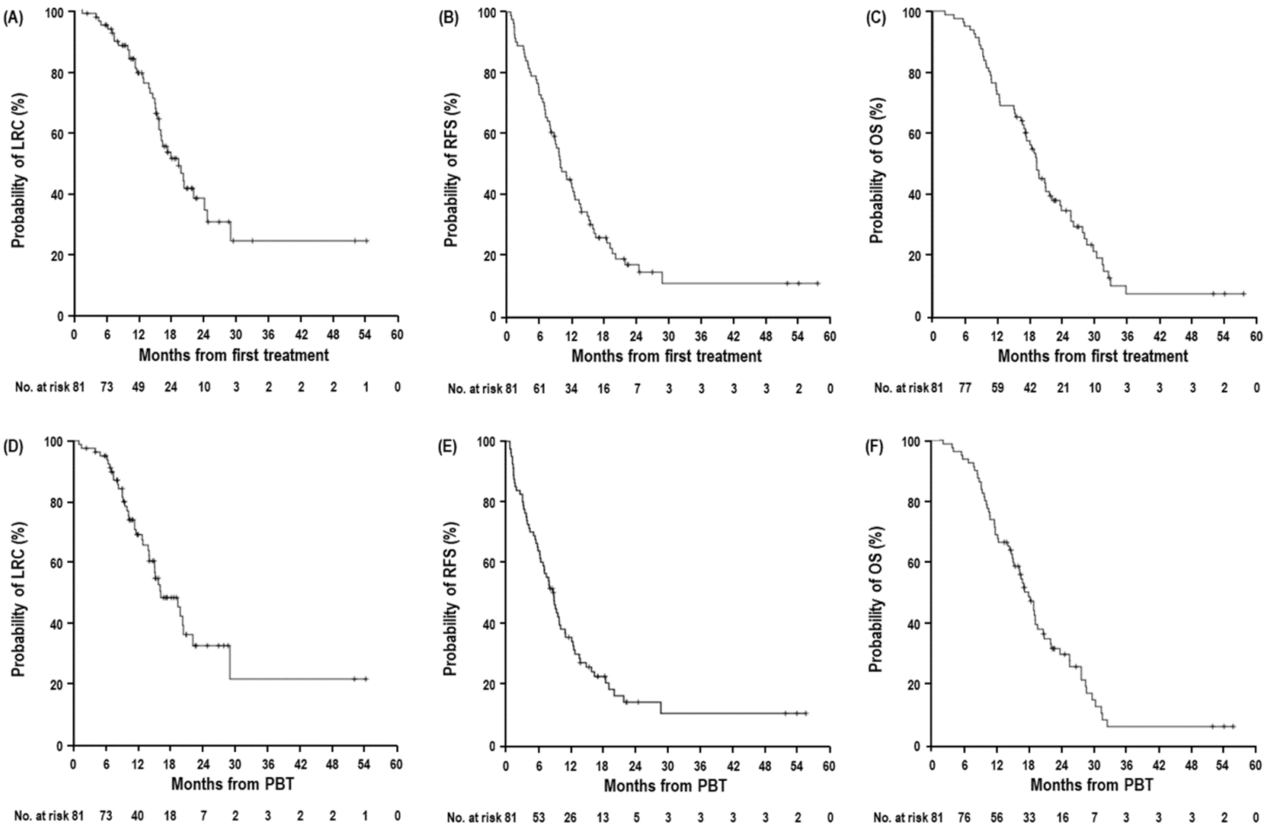

Supplementary Figure 1. Locoregional control (LRC) (A), progression-free survival (RFS) (B), and overall survival (OS) (C) curves from initial treatment in all patients and LRC (D), PFS (E), and OS (F) from PBT in all patients.
